# A first case of *Hepatozoon* infection in a wild Amami rabbit (*Pentalagus furnessi*) in Tokunoshima Island, Japan

**DOI:** 10.1016/j.ijppaw.2026.101209

**Published:** 2026-02-20

**Authors:** Tsumugi Saito, Honoka Noguchi, Toshihiro Tokiwa, Hisashi Yoshimura, Ryotaro Suzuki, Ryouta Torimoto, Taisei Shiraishi, Kana Matsumoto, Makoto Haritani, Masami Yamamoto

**Affiliations:** a Laboratory of Physiological Pathology, School of Veterinary Nursing and Technology, Nippon Veterinary and Life Science University, 1-7-1 Kyonan-cho, Musashino, Tokyo, Japan; b Laboratory of Veterinary Parasitology, School of Veterinary Medicine, Nippon Veterinary and Life Science University, 1-7-1 Kyonan-cho, Musashino, Tokyo, Japan; c Yuinoshima Animal Clinic, Nazeishibashi-cho, Amami, Kagoshima, Japan; d Amami Wildlife Conservation Center, Ministry of the Environment, Ongachi, Yamato-son, Oshima-gun, Kagoshima, Japan

**Keywords:** Amami rabbit, Apicomplexa, Endangered species, *Hepatozoon*, *Pentalagus furnessi*, UNESCO world heritage site

## Abstract

We report the first confirmed *Hepatozoon* infection in a wild Amami rabbit, *Pentalagus furnessi* (Lagomorpha: Leporidae), an endangered species (IUCN Red List) native to the Amami Archipelago in southwestern Japan. Necropsy was performed on an adult male that was rescued on Tokunoshima Island after a presumed vehicular injury and subsequently died. Histopathological examination revealed meronts in the pulmonary, myocardial, and skeletal muscle tissues without an associated inflammatory response. Transmission electron microscopy confirmed the presence of intracellular meronts at various stages of asexual development. Based on morphological and molecular analyses, the protozoan was identified as *Hepatozoon* sp. (Apicomplexa: Adeleorina). Phylogenetic analysis revealed that the isolate clustered within a monophyletic clade of *Hepatozoon felis*. The detection of asexual reproductive stages indicates that Amami rabbits can support merogony and is consistent with their role as intermediate rather than paratenic hosts in the *Hepatozoon* life cycle. These results highlight the need for further investigation of the ecology, transmission dynamics, and conservation implications of protozoan infections in endangered wildlife.

## Highlights

• First confirmed *Hepatozoon* infection in an endangered Amami rabbit (*Pentalagus furnessi*).
• Histopathology revealed meronts in the lungs, heart, and skeletal muscle tissues.
• Molecular and phylogenetic analyses showed a close relationship to *Hepatozoon felis.*
• Evidence of asexual reproduction suggests Amami rabbits may act as intermediate hosts.

## Graphical abstract

**Figure:**
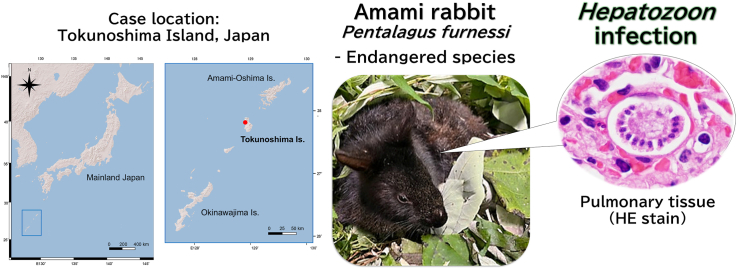

## 1 Introduction

Tokunoshima Island, part of the Amami Archipelago, is included in the UNESCO World Natural Heritage Site for its humid subtropical ecosystems and high level of endemism (UNESCO World Heritage Centre, 2021).

Among the island's many unique species, the Amami rabbit, *Pentalagus furnessi* (Lagomorpha: Leporidae), is a relict lineage. Endemic to both the Amami-Oshima and Tokunoshima Islands, it has been designated a Japanese Special Natural Monument owing to its deep evolutionary divergence and distinctive biological traits (Yamada, 2008). The species is currently listed as *Endangered* on the IUCN Red List (Yamada and Smith, 2016). Pellet count surveys conducted between 1992 and 1994 estimated that the Amami rabbit population on Tokunoshima Island was only 120–300 individuals (Sugimura et al., 2000; Yamada et al., 2000; Yamada, 2008). By 2021, this population had increased markedly to approximately 1525–4735 individuals, likely reflecting intensive control of invasive predators, particularly stray and feral cats (*Felis catus*) (Kyushu Regional Environmental Office, Ministry of the Environment, Japan, 2022). Nevertheless, long-term challenges remain, including habitat fragmentation and restricted genetic exchange (Yamada and Izawa, 2025).

Furthermore, a recent genetic study revealed that Amami rabbits in Tokunoshima Island possess distinct genetic markers and unique haplotypes that differentiate them from their counterparts in Amami-Oshima Island (Kinoshita et al., 2025). This genetic uniqueness, likely resulting from long-term isolation and localised adaptation, underscores the critical need for targeted conservation measures to preserve these unique genetic resources. Given these circumstances, further investigation of health threats, including infectious diseases and parasitism, is essential.

*Hepatozoon* is a genus of apicomplexan protozoan parasites infecting a wide range of vertebrate hosts (Yamamoto et al., 2017; Dubey and Baneth, 2025). *Hepatozoon* spp. can cause clinical manifestations ranging from subclinical infection to severe disease, particularly in immunocompromised hosts or in the presence of concomitant infections (De Bonis et al., 2021; Dubey and Baneth, 2025). However, knowledge of *Hepatozoon* infection in lagomorphs remains limited. In 1908, Patton reported an intraleukocytic parasite in Indian black-naped hares (*Lepus nigricollis*) that was later reclassified as *Hepatozoon leporis* (Bhatia, 1938; Nandi and Das, 2010). Sangiorgi (1914) described a related parasite in domestic rabbits, characterised by gamonts in leukocytes and large meronts in visceral organs, which is now recognised as *Hepatozoon cuniculi* (Pritt et al., 2012). Despite these early descriptions, independent confirmation of both species in recent decades has been limited, leaving their epidemiology and life cycle incompletely understood. Recent molecular surveys of *Hepatozoon* spp. have generally detected a low prevalence in rabbits and hares (Johnson et al., 2009; Uiterwijk et al., 2023).

*Hepatozoon* spp. are primarily transmitted through the ingestion of infected haematophagous arthropods, most commonly ticks, in which sexual development of the parasite occurs; transmission may also involve predation on infected intermediate or paratenic hosts, depending on the host–parasite system (Dubey and Baneth, 2025; Johnson et al., 2009). In the Amami rabbit, the host-specific tick *Haemaphysalis pentalagi* is known to occur (Kwak et al., 2024). In addition, several ixodid tick species have been recorded on Tokunoshima Island, including *Amblyomma testudinarium*, *Dermacentor taiwanensis*, *Haemaphysalis formosensis*, *H. hystricis*, *H. longicornis*, *Ixodes granulatus*, and *Rhipicephalus sanguineus* sensu lato (Fujita et al., 1996; Mikomoda et al., 2006). Although detailed information on their utilization of the Amami rabbit as a host remains unclear, and there are no records of *Hepatozoon* infection in these ticks to date, the presence of these tick species suggests that local tick fauna may provide an ecological basis for potential transmission cycles of *Hepatozoon* spp. in this ecosystem.

Therefore, documenting systemic *Hepatozoon* infection in the endangered Amami rabbit is important because reports of *Hepatozoon* in lagomorphs are rare and their roles as intermediate versus paratenic hosts remain uncertain (Johnson et al., 2009; Uiterwijk et al., 2023). The present report expands the known host range of *Hepatozoon* and provides valuable context for understanding transmission pathways and life cycle dynamics, with potential relevance to conservation-related disease ecology in insular wildlife populations.

## 2 Materials and methods

### 2.1 Case data and specimen collection

**Fig. 1:**
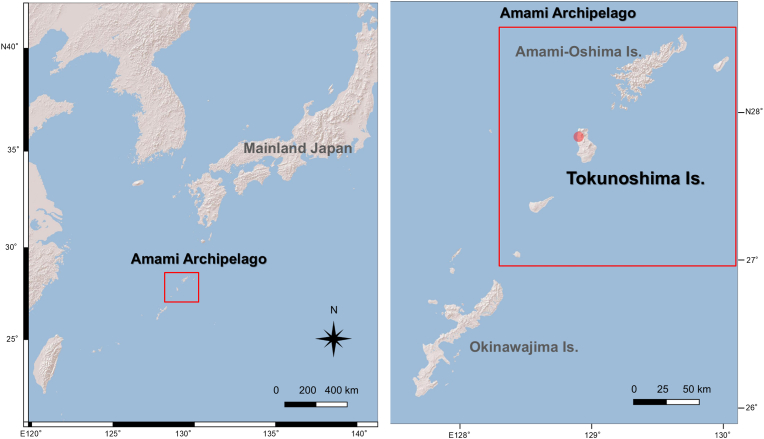
Map showing the location of the Amami Archipelago, including Tokunoshima Island, in southwestern Japan and the surrounding region. The red circle indicates the rescue site of the *Hepatozoon*-infected Amami rabbit.

We investigated a case of an adult male Amami rabbit. In December 2022, the animal was found crouched at the roadside in Amagi-cho, Tokunoshima Island, Kagoshima, Japan (Fig. 1), and was subsequently rescued. Although no apparent signs of external trauma were noted, the presence of hind limb paralysis suggests that the rabbit was likely struck by a vehicle. The animal was transported by air to an animal hospital on the neighbouring island, Amami-Oshima, but did not recover and died suddenly on the 17th day after admission.

During necropsy at the animal hospital, organs (brain, lumbar spinal cord, heart, trachea, lungs, liver, gallbladder, spleen, adrenal glands, kidneys, testicles, thigh muscles, oesophagus, stomach, jejunum, ileum, caecum, and colon) were fixed in 10% neutral-buffered formalin, and muscle tissues from the forelimbs, hind limbs, and dorsal regions were collected and fixed in 70% ethanol. The specimens were transported to the Nippon Veterinary and Life Science University in Tokyo for further examination.

### 2.2 Histopathological study

Tissue samples were fixed in 10% neutral-buffered formalin and processed for routine histopathology. The specimens were embedded in paraffin, and 4-μm-thick sections were prepared using a microtome. The sections were subsequently stained with haematoxylin and eosin (HE) and examined under a light microscope BX53 (Olympus, Tokyo, Japan) using a DP74 Microscope Digital Camera and cellSens software (Olympus) to evaluate tissue architecture and detect any parasitic stages or pathological lesions.

In addition to the present case, to evaluate the occurrence of protozoan developmental stages in Amami rabbits examined in our laboratory, we retrospectively reviewed HE-stained tissue sections from 67 necropsy cases submitted between 2017 and 2024 (64 from Amami-Oshima and 3 from Tokunoshima Islands). Sections of major organs were examined when available and the examined organs varied depending on the case.

### 2.3 Ultrastructural study

For ultrastructural analysis, selected areas of HE-stained lung tissue sections were identified as regions of interest. These areas were reprocessed using the inverted gelatine capsule method for resin embedding (Morecki and Becker, 1968). Ultrathin sections were prepared from resin-embedded tissues, stained with uranyl acetate and lead citrate, and examined using a transmission electron microscope JEM-1011 (JEOL, Tokyo, Japan) to assess the fine ultrastructural features.

### 2.4 Polymerase chain reaction and sequence analysis

DNA was extracted from alcohol-fixed skeletal muscle tissues collected from the hind limb and dorsal muscles using the QIAamp DNA Mini Kit (Qiagen, Hilden, Germany), according to the manufacturer's instructions. Polymerase chain reaction (PCR) amplification of a partial segment of the 18S rRNA gene (*18S*) was performed using primers specific for *Hepatozoon* species, 18S1F (5′-GGATAACCGTGGTAATTCTATG-3′) and 18S11R (5′-TCCTATGTCTGGACCTGGTGAG-3′) (Pritt et al., 2008). The PCR products were purified using a MinElute PCR Purification Kit (Qiagen) and subjected to direct sequencing (Eurofins Genomics, Tokyo, Japan). The obtained *18S* sequences were compared with sequences available in the GenBank database using the Basic Local Alignment Search Tool (BLAST; http://blast.ncbi.nlm.nih.gov/Blast.cgi), and a representative sequence was deposited in the DNA Data Bank of Japan.

### 2.5 Phylogenetic analysis

Phylogenetic analysis was performed based on *18S* sequences. A total of 61 sequences belonging to the suborder Adeleorina (Apicomplexa) were retrieved from the International Nucleotide Sequence Database Collaboration and multiple sequence alignments were conducted using MAFFT v7 (Katoh et al., 2019) with the Q-INS-i strategy, together with the sequences obtained in this study. A phylogenetic tree was constructed using the maximum likelihood method implemented in IQ-TREE (Trifinopoulos et al., 2016), under the TPM2u + F + R3 substitution model. *Adelina dimidiata* (Adeleidae) was used as an outgroup for the root tree. The branch support (BS) was assessed using an SH-like approximate likelihood ratio test (SH-aLRT) with 1000 replicates. The resulting tree was visualised and edited using iTOL v6 (Letunic and Bork, 2024).

## 3 Results

### 3.1 Histopathological findings

**Fig. 2:**
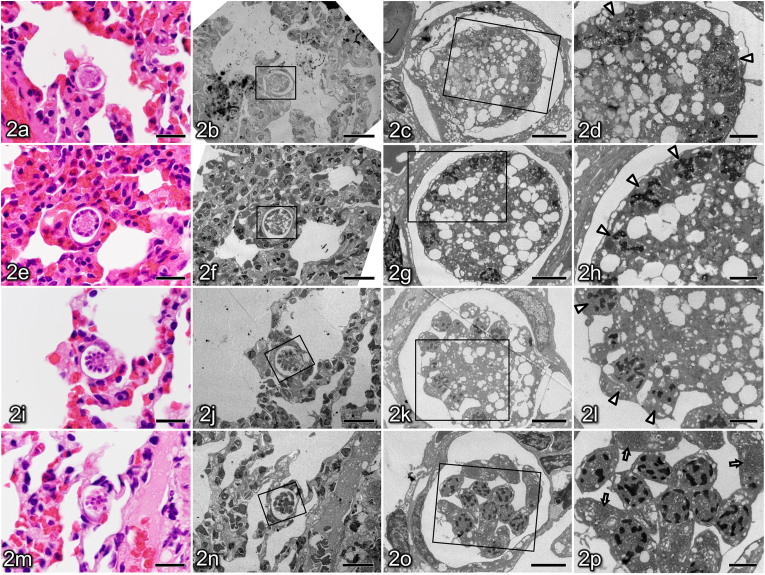
Histological and ultrastructural features of *Hepatozoon* meronts in the lung of an Amami rabbit, illustrating progressive meront development. Panels (a–d), (e–h), (i–l), and (m–p), represent early-, mid-, late-developing, and mature meronts, respectively. In each set, panel (a, e, i, m) shows the haematoxylin and eosin-stained section, and panels (b–d, f–h, j–l, n–p) show corresponding transmission electron microscopy images of the same meront region at sequentially increasing magnification, obtained using the inverted gelatine capsule method. Boxes indicate the areas enlarged in the subsequent panels. Early developing meront (a–d) with a few round nuclei (arrowheads) located at the periphery of a vacuolated cytoplasm. Mid-developing meront (e–h) showing an increased number of peripheral nuclei (arrowheads). Late-developing meront (i–l) in which the peripheral nuclei (arrowheads) begin to separate from the central cytoplasm. Mature meront (m–p) containing crescent-shaped merozoites with electron-dense granules (arrows) within the cytoplasm. Scale bars: 20 μm (a, b, e, f, i, j, m, n), 5 μm (c, g, k, o), and 2 μm (d, h, l, p).

In the present case, histopathological examination revealed that meronts were frequently observed in the cells of the alveolar walls of the lung. These meronts were present at various developmental stages, including the early (Fig. 2a), mid-developing (Fig. 2e), late developing (Fig. 2i), and mature meronts (Fig. 2m). Twenty meronts that appeared to be sectioned at their maximal diameter were randomly selected and measured, yielding dimensions of 20.3 ± 2.4 × 16.2 ± 2.0 μm in diameter, excluding the capsule. The capsule width was 1.1 ± 0.3 μm. In the heart, meronts were sporadically observed within myocardial cells. In thigh muscle, meronts were rarely detected within skeletal muscle cells or in the interstitial cells between muscle fibres. In all the organs where meronts were detected (heart, lungs, and thigh muscles), no inflammatory reaction was observed surrounding them.

In the retrospective review of 67 necropsy cases, such meronts were identified histologically in only one case, which was the present case.

### 3.2 Ultrastructural analysis

Ultrastructural analysis of lung tissue using transmission electron microscopy revealed meronts at various stages of maturation within host cells. Early developing meronts within a membrane-bound parasitophorous vacuole exhibited a wide cytoplasm containing numerous amylopectin granules and coarse empty vacuoles. A small number of nuclei containing electron-dense chromatin were found mainly in the cytoplasmic periphery (Fig. 2b–d). In mid-developing meronts, the number of nuclei increased, and they were arranged more peripherally than in the immature stage (Fig. 2f–h). Late-developing meronts showed initiation of cytoplasmic segmentation. Incomplete merozoites with a single nucleus often arranged toward the periphery of the meront, leaving a central residual cytoplasmic area. (Fig. 2j–l). In mature meronts, elongated oval- or crescent-shaped merozoites produced by asexual proliferation were observed, and numerous electron-dense micronemes approximately 100 nm in diameter and a few rhoptries were observed within the cytoplasm (Fig. 2n–p). The merozoite measured approximately 2.7 × 8.7 μm.

### 3.3 PCR and sequence analysis

PCR analysis demonstrated that the target band was detected in both the tested samples. Sequence analysis revealed that the amplified sequences from both samples were identical, confirming the presence of *Hepatozoon* spp. The representative 1028-bp sequence was deposited in the database under accession number LC877788. The *18S Hepatozoon* sequence obtained from *P. furnessi* in the present study showed high similarity (99.51% identity with 100% query cover) to a *H. felis* sequence from *Felis silvestris* from Hungary (OM422756).

**Fig. 3:**
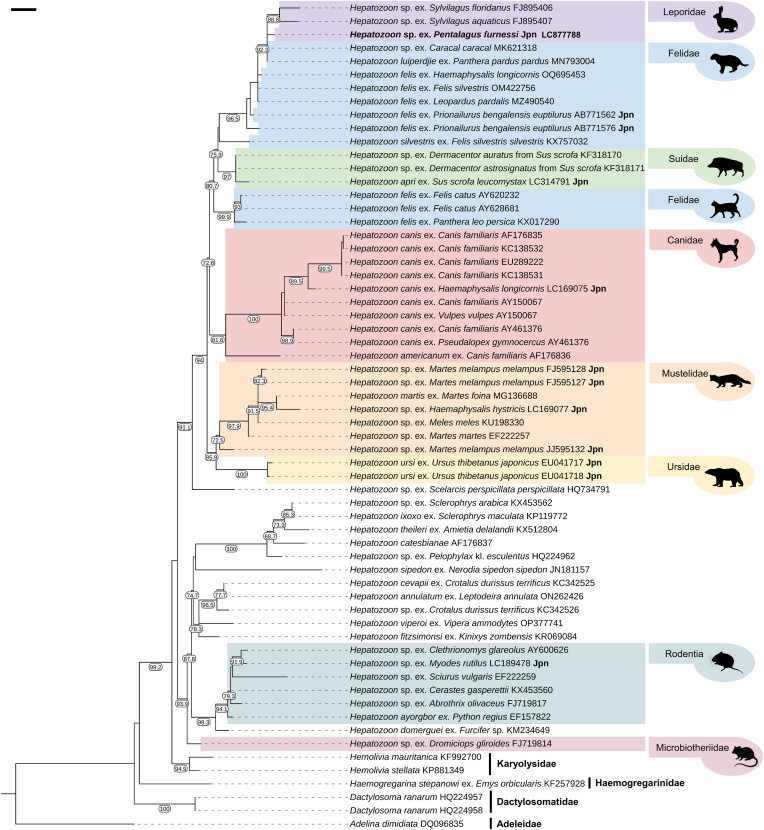
Maximum-likelihood phylogenetic tree of *Hepatozoon* and other members of Adeleorina (Karyolysidae, Haemogregarinidae, Dactylosomatidae, and Adeleidae) based on *18S* sequences. Nodal support values represent SH-aLRT support values (≥65). Species name, host, and accession number are shown on the right side on the tree. “Jpn” indicates the sequences detected in Japan. The scale bar represents 0.1 substitution per site.

In the phylogenetic tree based on *18S* sequences, the genus *Hepatozoon* formed a monophyletic group (Fig. 3). The *Hepatozoon* clade was divided into two major clades with high support values (BS = 91.1–93.9): one clade consisting of sequences detected from carnivores, wild boar, and lagomorphs, and another clade comprising sequences from rodents, reptiles, and amphibians. The former clade was further divided into two subclades: one comprising sequences detected from Ursidae and Mustelidae (BS = 85.9) and the other from Felidae, Canidae, Suidae, and Leporidae (BS = 72.8). *Hepatozoon canis* and *H. americanum*, both of which are detected in Canidae, formed a monophyletic group (BS = 81.6). In contrast, the clade comprising sequences of *H. felis*, *H. silvestris*, and *H. lupiperdjie* from Felidae also included *H. apri* detected in *Sus scrofa* (Suidae) and an unidentified *Hepatozoon* sp. from Leporidae. Notably, *H. felis* was polyphyletic and comprised two distinct lineages: one including sequences from *Panthera leo persica* and *Felis catus* (BS = 99.9), and the other including sequences from *F. silvestris*, *Prionailurus bengalensis euptilurus, Leopardus pardalis,* and *Panthera pardus pardus*. The sequence of *Hepatozoon* sp. obtained from *P. furnessi* in this study formed a monophyletic group with *Hepatozoon* sp. sequences detected from *Sylvilagus* spp. (Lagomorpha: Leporidae) in the USA (FJ895406, FJ895407), *Panthera pardus pardus* (Carnivora: Felidae) (MN793004), and *Caracal caracal* (Carnivora: Felidae) (MK621318) in South Africa (BS = 92.3).

## 4 Discussion

This is the first confirmed case of *Hepatozoon* infection in an Amami rabbit. Among 67 necropsy cases, meronts were histologically identified only in the present case. A separate histological survey of 131 archival specimens (126 from Amami-Oshima and 5 from Tokunoshima Islands) conducted between 2003 and 2012 also found no evidence of infection (Kubo et al., 2013). Together, these findings suggest that such infections are uncommon in this species.

*Hepatozoon* infection in lagomorphs has been reported only sporadically, and its epidemiology remains poorly understood. Prevalence data suggest that *Hepatozoon* infections in lagomorphs are rare: *Hepatozoon* DNA was not detected in 12 wild European hares in Spain (Gimenez et al., 2009), and only 1 of 171 hares (0.6%) in Croatia tested PCR-positive for *H. canis* (Uiterwijk et al., 2023), possibly reflecting indirect transmission from canids. Despite their apparent rarity, lagomorphs may play epidemiologically important roles in certain parasite systems; for example, in *Hepatozoon americanum*, rabbits serve as paratenic hosts that facilitate transmission to canids via predation (Johnson et al., 2009).

In our study, molecular analysis revealed that the *Hepatozoon* species detected in the Amami rabbit formed a monophyletic group with sequences registered as *H. felis*, a species typically found in domestic cats. Although *Hepatozoon* species have traditionally been considered host-specific, recent molecular studies have suggested that many species can infect multiple host species (Thomas et al., 2024). Such apparent host plasticity further complicates species-level identification when based solely on the host species from which the *Hepatozoon* was detected and on *18S*-based phylogenetic analyses. *Hepatozoon felis* is generally regarded as having a high degree of host specificity for felids, with confirmed infections in domestic cats and wild species, such as the European wildcat (*Felis silvestris*) and tiger (*Panthera tigris*) (Grillini et al., 2023). However, *H. felis*-like sequences have also been reported in non-felid hosts, including Pampas foxes (*Lycalopex gymnocercus*), South American grey foxes (*L. griseus*), and spotted hyenas (*Crocuta crocuta*) (Giannitti et al., 2012; Williams et al., 2014; Millán et al., 2019). Additionally, *H. felis*-like sequences have been identified in ticks (*Amblyomma fimbriatum*) collected from a yellow-spotted monitor (*Varanus panoptes*) in Australia and in rodents (*Rattus rattus* and *R. norvegicus*) in Nigeria (Vilcins et al., 2009; Kamani et al., 2018). Whether these non-felid hosts were infected with *H. felis* or a closely related but distinct *Hepatozoon* species remains unclear.

Allen et al. (2011) reported *Hepatozoon* sequences detected in the heart tissue of eastern cottontail rabbits (*Sylvilagus floridanus*) and swamp rabbits (*S. aquaticus*) in Oklahoma, USA. Interestingly, phylogenetic analysis based on *18S* sequences revealed a close evolutionary relationship between the *Hepatozoon* genotypes found in these lagomorphs and those found in felids (Thomas et al., 2024). These findings led the authors to consider, as one possible hypothesis, that rabbits may serve as paratenic hosts for *Hepatozoon* spp. related to those found in felids. However, this study did not clarify whether *Hepatozoon* undergoes asexual reproduction in the rabbit host.

To the best of our knowledge, TEM-based ultrastructural observations of *Hepatozoon* in lagomorphs have not been reported. Nevertheless, the merozoites in the present case contained small electron-dense cytoplasmic granules, a finding compatible with invasive apicomplexan zoites. More importantly, we captured a peripheral budding (rosette-like) pattern of merozoite formation along the meront periphery with a residual central body, which is regarded as a characteristic mode of merogony in *Hepatozoon* (Dubey and Baneth, 2025). Together, these observations provide ultrastructural support for active asexual development within a lagomorph host and support the role of the Amami rabbit as an intermediate host in the *Hepatozoon* life cycle.

Although ingestion of infected ticks is considered a major route of *Hepatozoon* transmission (Dubey and Baneth, 2025), we incidentally observed ticks in the intestinal tract of a separate necropsied Amami rabbit (data not shown), suggesting that oral ingestion of ticks (e.g., during grooming) may occur. Further studies incorporating additional genetic markers and broader sampling of potential hosts and vectors on Tokunoshima Island will be necessary to clarify the transmission cycle of the *H. felis*-like lineage detected in the Amami rabbit.

Finally, the present study provides the first confirmed evidence of infection by a *Hepatozoon* species closely related to *H. felis* in a wild Amami rabbit, thereby expanding our understanding of host–parasite interactions in lagomorphs. Histopathological examination revealed *Hepatozoon* meronts in the lungs, heart, and skeletal muscles without any accompanying inflammatory response, suggesting that the infection was not associated with overt disease in this host. From a conservation perspective, the detection of systemic *Hepatozoon* infection in this endangered, island-endemic lagomorph warrants attention even in the absence of overt pathology. Subclinical infections may still affect host fitness under environmental stressors or in combination with other infections, and continued surveillance will be important to assess prevalence, transmission dynamics, and any potential health impacts on the Tokunoshima population.

## CRediT authorship contribution statement

**Tsumugi Saito:** Writing – original draft, Visualization, Investigation. **Honoka Noguchi:** Writing – original draft, Visualization, Investigation. **Toshihiro Tokiwa:** Writing – review & editing, Visualization, Validation, Methodology, Formal analysis. **Hisashi Yoshimura:** Writing – review & editing, Writing – original draft, Visualization, Project administration, Methodology, Funding acquisition, Formal analysis, Data curation, Conceptualization. **Ryotaro Suzuki:** Validation, Project administration, Investigation, Data curation. **Ryouta Torimoto:** Resources, Investigation. **Taisei Shiraishi:** Resources, Investigation. **Kana Matsumoto:** Visualization, Investigation. **Makoto Haritani:** Writing – review & editing, Visualization, Validation, Supervision, Investigation. **Masami Yamamoto:** Validation, Supervision, Project administration, Methodology, Conceptualization.

## Declaration of generative AI and AI-assisted technologies in the manuscript preparation process

During the preparation of this work, the authors used *ChatGPT (OpenAI)* in order to assist with language editing. After using this tool, the authors reviewed and edited the content as needed and take full responsibility for the content of the published article.

## Declaration of competing interest

The authors declare that they have no competing interests or personal relationships that could have appeared to influence the work reported in this paper.
